# Callous-Unemotional Traits and Antisocial Behavior in South Korean Children: Links with Academic Motivation, School Engagement, and Teachers’ Use of Reward and Discipline

**DOI:** 10.1007/s10802-020-00663-2

**Published:** 2020-06-16

**Authors:** Suhlim Hwang, Rebecca Waller, David J. Hawes, Jennifer L. Allen

**Affiliations:** 1 Department of Psychology and Human Development, UCL Institute of Education, London, UK; 2 Department of Psychology, University of Pennsylvania, Philadelphia, USA; 3 School of Psychology, University of Sydney, Sydney, Australia; 4 Department of Psychology, University of Bath, Bath, UK

**Keywords:** Callous-unemotional traits, Psychopathic traits, Teacher-child interaction, Discipline, Academic motivation, School engagement

## Abstract

Callous-unemotional (CU) traits have been associated with atypical responses to reward and punishment cues, with evidence suggesting that such traits may shape caregiver use of reward and punishment practices over time. To date, research has predominantly focused on parental rewards and discipline, with far less attention paid to teacher behavior management strategies. The first aim of the current study was to investigate the potential moderating effect of CU traits on the relationship between teacher classroom management strategies (rewards and discipline) and two important school-related outcomes: student engagement and academic motivation. The second aim was to examine whether CU traits were related to teachers’ use of discipline and reward strategies over time. Children attending South Korean primary schools (*N* = 218; aged 10–12 years; 52% boys) reported on CU traits, antisocial behavior, teacher classroom management strategies, school engagement and academic motivation at two time points (the beginning and end of a single academic year). First, harsh teacher discipline predicted lower school engagement, but only for children low in CU traits. Second, cross-lagged longitudinal models showed that CU traits predicted decreased use of teacher rewards, over and above associations with antisocial behavior. CU traits were not related to harsh discipline cross-sectionally or longitudinally in models that accounted for antisocial behavior. Findings show that CU traits are related to reduced sensitivity to teacher discipline, suggesting that teachers may need additional support to implement both discipline and reward-based strategies with children high in these traits.

**Electronic supplementary material:**

The online version of this article (10.1007/s10802-020-00663-2) contains supplementary material, which is available to authorized users.

Antisocial behavior represents a major challenge to schools because of its substantial negative impact on students and teachers. Disruptive classroom behavior is a major reason for teacher stress and burnout, with pupil misbehavior preventing teachers from maintaining a harmonious classroom environment (Westling 2010). Antisocial behavior predicts poor school outcomes, including bullying, truancy, school exclusion, school dropout, and poor academic achievement (Carroll et al. 2009). Increasing disengagement and failure at school, often acting in combination with antisocial peer group involvement and a challenging home environment, may steer at-risk children towards a trajectory of antisocial behavior (Allen et al. 2020). The temperament dimension of callous-unemotional (CU) traits, characterized by reduced empathy, lack of guilt or remorse, shallow emotions and a lack of concern for performance, has shown substantial utility in explaining differing pathways to antisocial behavior (Waller et al. 2019). CU traits predict more severe and persistent antisocial behavior and are associated with distinct socioemotional correlates, such as reduced recognition of and responding to others’ distress cues (Marsh and Blair 2008). CU traits are characterized by a shallow, self-serving motivational set coupled with a lack of concern about the potential consequences of antisocial behavior, including anticipated feelings of guilt, disciplinary action, or victim distress (Foulkes et al. 2014). Children with CU traits do not appear motivated by social approval or affiliation, reflected in their poor quality relationships with significant others (Waller and Wagner 2019).

To date, the bulk of research on CU traits and their influence on social interaction has focused on parenting, leading to calls for greater attention to CU traits in the school context to help guide school-based interventions (Tyler et al. 2019). In terms of the differential impact of punishment versus reward-based parenting practices on CU traits, early studies suggested that harsh parental discipline predicted more severe antisocial behavior among children with low, but not high, levels of CU traits (i.e., a significant moderation effect), suggesting that children with high CU traits may be less sensitive to parental discipline (Hipwell et al. 2007; Oxford et al. 2003). However, later longitudinal evidence suggested that parents may increase the harshness of their discipline when their child is less responsive to negative reinforcement, but in doing so exacerbate risk for CU traits and antisocial behavior (Waller et al. 2015; Waller et al. 2016; Waller et al. 2017). Other longitudinal studies have reported that higher levels of positive parenting are associated with reduced risk for antisocial behavior regardless of the level of children’s CU traits (Hyde et al. 2013; Waller et al. 2015), and even that greater use of positive parenting practices predicts decreases in antisocial behavior specifically among children with elevated levels of CU traits (Clark and Frick 2018; Kochanska et al. 2013). Finally, research has also begun to establish direct, and potentially reciprocal, associations between CU traits and parenting. For example, in a study of children aged 3 to 10 years, CU traits predicted more negative parenting (Hawes et al. 2011) and in 2–3 year old children, lower parental warmth predicted increases in CU traits while high CU traits simultaneously predicted decreases in parental warmth (Waller et al. 2014). Reciprocal associations were also found in an adoption study of children aged 1.5–4.5 years, where CU traits predicted increases in harsh parenting while harsh parenting simultaneously predicted increases in CU traits (i.e., ruling out passive-gene environment correlations; Trentacosta et al. 2019). Overall, findings from parenting studies suggest that 1) CU traits may differentially impact child responsiveness to parenting practices, and 2) parents may escalate their use of coercive control strategies and decrease rewards when these strategies do not obtain the desired results.

By the age of around 5, most children start formal schooling and spend more time outside of the home, typically spending up to 6.5 hours a day in a classroom (OECD 2019). Despite the importance of the school context for child development, studies have rarely investigated the role of teacher socialization in the etiology of CU traits, nor its potential impact on school outcomes. Like parenting practices, teacher classroom management strategies draw on social learning theory principles to emphasize the role of rewards (e.g., praise) in promoting prosocial behavior and engagement with schoolwork, as well as calm, consistent non-physical discipline (e.g., loss of privileges) to deal with antisocial behavior (Webster-Stratton 2001). Indeed, classroom management strategies are one of the most influential factors affecting student motivation, learning and behavior (Wang et al. 1993). Greater use of rewards is related to children’s positive development and academic skills (Sutherland, et al. 2000), whereas coercive discipline is associated with increased antisocial behavior and disengagement from school (Lewis, et al. 2005). Teacher-child interaction comprises sequences of reciprocal, mutually reinforcing interactions, where the coercive behaviors of children (e.g., refusing to work, aggravating classmates) and teachers (e.g., shouting, criticism) can serve to maintain and escalate child antisocial behavior (Sutherland and Morgan 2003). Indeed, teachers employ harsh and inconsistent discipline strategies more frequently with antisocial children, leading to even greater student disengagement and noncompliance with teacher requests (Nelson and Roberts 2000; Skinner and Belmont 1993). Conversely, teacher reward strategies help to establish a supportive classroom environment, and to decrease aggression and non-compliance in antisocial children (Moffat 2011).

Preliminary evidence suggests that CU traits cause significant impairment in the school setting, including low levels of school connectedness (Fanti et al. 2017), poor relationships with teachers (Horan et al. 2016), and poor academic achievement (Bird et al. 2019). While verbal ability deficits are considered to be a major contributing factor to school failure in antisocial children (Moffitt 1993), evidence suggests that these deficits are unrelated to CU traits (Allen et al. 2013; DeLisi et al. 2011). It has therefore been suggested that rather than reduced cognitive abilities, the poor achievement of antisocial children with CU traits may be due to low intrinsic motivation and school engagement, coupled with reduced responsiveness to teacher strategies (Allen et al. 2018; DeLisi et al. 2011; Horan et al. 2016). However, only a handful of studies have explored the relationship between CU traits, children’s responses to teachers’ use of reward and discipline strategies, and school-related outcomes. Two qualitative studies found that teachers perceived both rewards and discipline as less effective overall for children high in CU traits; but praise and a positive teacher-child relationship were perceived as helpful to promote academic motivation and school engagement, while sending children out of class was also considered to be effective for reducing disruptive behavior (Allen et al. 2018; Allen et al. 2016). Researchers have also suggested the possibility of a reciprocal relationship, as children with CU traits may not derive the same benefits from teacher strategies that promote motivation, engagement and prosocial behavior in typically developing children, and conversely, callous-unemotional features may elicit harsher discipline, fewer rewards and less encouragement from teachers (Bird et al. 2019; DeLisi et al. 2011; Horan et al. 2016). However, prior studies are cross-sectional, prohibiting an examination of the direction of effects between teacher strategies and child outcomes. Therefore, the aim of the current study was to investigate longitudinal associations between CU traits and teacher strategies in relation to children’s school engagement and academic motivation. Note that motivation and engagement have sometimes been used interchangeably (Martin 2007), with motivation considered a pre-requisite element for engagement as an action (Fredericks, Blumenfeld, & Paris, 2004). However, others have argued that motivation is not sufficient for engagement, and that they should be considered distinct constructs (Eccles and Wang 2012). The present study therefore explored both motivation and engagement, modelling these constructs as correlated outcomes to parse their unique relationship with CU traits while accounting for their conceptual overlap.

The current study is the first to explore longitudinal relationships between CU traits, teacher discipline and reward strategies, and school outcomes (intrinsic academic motivation, school engagement). In particular, we conducted this study across a full academic year in order to capture the practices of the same classroom teacher’s behavior at multiple time points. From a developmental perspective, the study focused on a period when children in South Korea have intensive contact with their teacher throughout the school year (primary school years 5 and 6), and a time known to coincide with a dramatic increase in children’s antisocial behavior (Kim et al. 2013). From a cross-cultural perspective, research on CU traits in East Asian nations is vital given that past research has revealed cultural differences in the presentation of CU traits, as well as the influence of social factors, such as parenting (Fung et al. 2009; Sng et al. 2018). Furthermore, findings from research in Western countries on CU traits and teacher-child interaction may not be generalizable to East Asian countries due to differing cultural values, along with differences in education policy, systems and teacher training (Cheng et al. 2004). Finally, previous studies have predominantly relied on teacher views, which may suffer from self-presentation biases regarding their use of reward and discipline strategies (Ciucci et al. 2014; Waschbusch and Willoughby 2008). Child report can provide useful information about children’s own perspective on their school functioning and the factors that reduce or increase their problematic behavior in the school context. To the best of our knowledge, the present study is the first to examine child perspectives on CU traits and their association with teacher behavior in the classroom.

We first investigated the potential moderating effect of CU traits on the relationship between teacher reward and discipline strategies at time 1 and both school engagement and intrinsic academic motivation at time 2. We hypothesized that CU traits would moderate the association between teacher harsh discipline and later school-related outcomes, such that harsh discipline would predict poor outcomes (low motivation, reduced engagement) in children with low but not high levels of CU traits. We also hypothesized that CU traits would moderate the association between teacher reward strategies and school outcomes, such that greater use of rewards by teachers would predict increased engagement and motivation among children with high, but not low levels of CU traits. Our second aim was to explore longitudinal reciprocal effects between CU traits and teacher’s use of both reward and discipline strategies using cross-lagged panel models. As before, co-occurring levels of antisocial behavior were accounted for in models to test for specificity in the relationships with CU traits. We hypothesized that CU traits would account for unique variance in teacher’s use of reward and discipline strategies over time, such that higher levels of CU traits would elicit fewer instances of reward strategies by teachers and more instances of harsh discipline.

## Method

### Participants

Participants were students aged 10 to 12 years from two public primary schools in a large south-eastern city of South Korea. Students were in Year 5 and 6, the two highest grades in the South Korean primary school system. In total, 218 students across 11 classrooms (Year 5, *n* = 3; Year 6, *n* = 8) were recruited. At the time 1 assessment, the sample had a mean age of 11.03 years (*SD* = 0.65; 52% male). All children were Korean and a small percentage (*n* = 21, 10%) were eligible for free school milk, an index of low socioeconomic status. A small proportion of the sample (*n* = 17, 8%) reported living with a single parent, largely consistent with the 10.9% reported single parent family rate in South Korea (Statistics Korea 2017). Of the 218 students who participated at the time 1, 214 students (98%) were retained at the time 2 assessment completed 9 months later. Students who were lost to follow-up did not differ on gender, membership of a single parent family, or any of the main study variables (range, *t* = 0.42–1.14, all *p*s > 0.05). Teacher participants included 11 homeroom teachers and their ages ranged from 30 to 55 years (M = 40.45, *SD* = 6.39, 36% male). To establish the convergent validity of measures, teachers reported on the CU traits and antisocial behavior of the four students from their classroom whom they judged to exhibit the most severe behavior problems, resulting in a subsample of 44 students.

#### Procedure

All study procedures and questionnaires were approved by the University College London Institute of Education ethics board prior to data collection. Permission to approach students to participate was sought through an invitation letter to the school principal. The parents of children in Year 5 and 6 were then informed of the research aims and procedures (*n* = 274) and parental opt-out consent was obtained. Twenty percent of parents (*n* = 56) opted out of consent, therefore only the remaining 218 students were approached to participate in the study. Child assent was sought on the day of the assessment, and all agreed to participate. Students completed the written questionnaires in their classroom during study hall time under exam conditions, and children who did not participate (20%) had study hall time as usual. Participants were given the option of not completing questions or returning an incomplete questionnaire. The first assessment was conducted in March 2018, at the start of the child’s new academic year and follow-up data was collected approximately 9 months later, in December 2018. In South Korea, winter vacation runs from late December to late January, and after winter vacation schools have reduced hours until the term ends in February. December is considered to be the end of the academic year, and the last academic assessment is also conducted then.

### Measures

#### Demographic Information

A brief child-report questionnaire assessed child age, gender (1 = female, 0 = male) at the time 1 assessment. As indices of socioeconomic status, teachers reported on family composition (1 = single parent, 0 = two parent) and the receipt of free school milk (1 = yes, 0 = no).

#### Child CU Traits and Antisocial Behavior

CU traits and antisocial behavior were assessed using available items from the child-report versions of the Strengths and Difficulties Questionnaire (SDQ; Goodman 1997) and the Antisocial Process Screening Device (APSD; Frick and Hare 2001). Both measures are rated on a 3-point scale from 0 (Not true) to 2 (Certainly true). Previously, the University of New South Wales (UNSW) system (Dadds et al. 2005) was developed via the pooled items of the SDQ and the APSD in order to enhance the specificity and validity of the assessment of CU traits in children. The reliability and construct validity of the UNSW CU traits and antisocial behavior index has been supported in many studies including samples of children and adolescents with alphas ranging from 0.69 to 0.89 with scores shown to correlate with child antisocial behavior (e.g., Hawes et al. 2019; Pasalich et al. 2011). Given that data for child self-report and Asian countries is less abundant regarding the UNSW system, we examined the validity of this model using confirmatory factor analysis (CFA). Specifically, CU traits were assessed using 5 items from the SDQ measuring prosocial behavior, or lack thereof (e.g., ‘inconsiderate of other people’s feelings’, ‘helpful if someone is hurt, upset, or ill’), as well as 4 items from the APSD assessing callousness (e.g., ‘concerned about the feelings of others’) and lack of guilt (e.g., ‘feel bad or guilty when you do something wrong’). Similarly, antisocial behavior was assessed using 3 items from the SDQ that tapped into aggression and rule-breaking (e.g., ‘often fights with other children’, ‘steals from home and elsewhere’) and 6 items from the APSD that assess broader symptoms of externalizing disorders (e.g., ‘gets bored easily’, ‘blames others’). We used CFA to assess the fit of a model specifying items to load onto separable CU traits and antisocial behavior factors as described above. At both time points, a two-factor model showed good model fit (Time 1, CFI = 0.90 and RMSEA = 0.05; Time 2, CFI = 0.95 and RMSEA =0.04) and antisocial behavior and CU traits were related to each other at both time points (Time 1, *β* = 0.35, *p* < 0.001; Time 2, *β* = 0.48, *p* < 0.001) suggesting that the items formed distinct, albeit related, constructs (See Table S1 for factor loadings and model fit statistics). In addition, both scales were not significantly associated with child age or free school milk, supporting the discriminant validity of the revised measures. The internal consistency of the CU traits scale in the current sample was 0.73 at time 1 and 0.74 at time 2. The internal consistency of the antisocial behavior scale was 0.72 at time 1 and 0.68 at time 2. Teacher-reported versions of the same items were used among a subsample of students (*n* = 44). The convergent validity of teacher and child report on the revised scales was supported by significant correlations between CU traits and antisocial behavior (*range*, *r* = 0.32–0.70, *p*s < 0.05) in a subsample of 44 students. Alphas for teacher report of CU traits and antisocial behaviour were 0.78 and 0.88, respectively.

#### Teacher Reward and Harsh Discipline Strategies

The classroom discipline strategies questionnaire (Lewis 2001) was used to obtain child report of teacher reward and harsh discipline strategies at each wave. Children rate the frequency of teachers’ use of rewards and harsh discipline strategies on a 5-point scale from 1 (never) to 5 (always). The reward dimension consisted of 8 items assessing how frequently teachers respond to good behavior from students (e.g., ‘reward individual students who behave properly’, ‘praises the class for good behavior’). The harsh discipline scale includes 5 items that assess teacher’s use of harsh discipline in response to child misbehavior (e.g., ‘yells angrily at students who misbehave’, ‘deliberately embarrasses students who misbehave’). The reliability and validity of the scale was supported in previous studies that included both elementary school students (Lewis 2001) and secondary school students (De Jong et al. 2013) with alphas ranging from 0.77 to 0.90 and scales showing significant relations with child disruptive behavior in the classroom. In the current sample, the alphas for reward strategies were 0.70 at time 1 and 0.81 at time 2. The alphas for harsh discipline were 0.75 at time 1 and 0.76 at time 2.

#### Intrinsic Academic Motivation

The intrinsic academic motivation scale of the Elementary School Motivation Scale (ESMS; Guay et al. 2010) was used to assess the pleasure and satisfaction that children derive from schoolwork. Children rated 9 statements on a 6-point Likert scale from 1 (always no) to 5 (always yes) (e.g., ‘I like reading’, ‘Writing interests me a lot’, ‘I do maths even when I do not have to’). The reliability of the ESMS were supported in previous studies (Garon-Carrier et al. 2016; Guay et al. 2017) with alphas ranging from 0.70 to 0.81 and scales showing associations with teacher instruction methods and child achievement. In the current sample, alphas for the academic motivation scale were 0.83 at time 1 and 0.89 at time 2.

#### School Engagement

The School Engagement Scale (SES; Fredricks et al. 2005) assessed child report of school engagement via 19 items that asked about participation in school activities, feelings toward school, and psychological investment in learning (e.g., ‘I pay attention in class’, ‘My classroom is a fun place to be’, ‘I read extra books to learn more about things we do in school’). Children rate each item on a 5-point scale from 1 (never) to 5 (all of the time). This measure has shown good reliability with alphas in previous studies ranging from 0.72 to 0.90, and scores on the SES have also shown strong relationships with teacher/peer support and child satisfaction (Göbel and Preusche 2019; Yang et al. 2020). In the current sample, alpha was 0.92 at time 1 and 0.93 at time 2.

### Data Analysis

Prior to analysis, descriptive statistics were explored (Table 1). We then examined bivariate correlations among the main study variables. To examine Aim 1, we used regression analysis and explored whether CU traits moderated the relationship between teacher strategies at time 1 and child school-related outcomes at time 2. The main effects (reward strategies, harsh strategies, and CU traits at time 1) and interaction effects (‘reward strategies × CU traits’ and ‘harsh strategies × CU traits’) were entered simultaneously. We examined the outcomes of child academic motivation and school engagement at time 2 as correlated dependent variables in one single regression model to parse the uniqueness of any effects of CU traits or teacher strategies. To account for autoregressive effects, we controlled for earlier academic motivation and school engagement at time 1, as well as the following covariates: child age, gender, family type, free school milk, and antisocial behavior. To probe the significant interactions, the association between teacher strategies and child school-related outcomes were examined at low and high levels of CU traits (1 SD below the mean and 1 SD above the mean, respectively), and we tested whether each slope of the regression lines differed significantly from zero (Cohen et al. 2003).

**Table 1:** Descriptive Statistics for the Main Study Variables (*N* = 218)

Variable	N	M	SD	Range	Skewness	Kurtosis
CU Traits Time 1	214	6.68	3.01	0–18	0.17	0.39
CU Traits Time 2	211	5.92	2.95	0–16	0.07	−0.39
AB Time 1	213	2.59	2.47	0–17	1.7	5.39
AB Time 2	211	2.72	2.30	0–10	0.83	0.30
Teacher Strategies						
Rewards Time 1	212	19.29	3.79	10–25	−0.20	−0.62
Rewards Time 2	212	19.17	3.73	8–25	−0.07	−0.50
Harsh Discipline Time 1	213	10.93	3.95	5–25	0.58	0.16
Harsh Discipline Time 2	212	11.67	4.24	1–23	0.26	−0.24
Academic Motivation Time 1	217	26.66	6.96	9–45	−0.06	0.32
Academic Motivation Time 2	213	26.37	8.06	9–45	−0.02	−0.18
School Engagement Time 1	214	66.53	12.53	23–95	−0.18	0.66
School Engagement Time 2	211	64.63	12.83	26–95	−0.26	0.59

*CU* traits Callous-unemotional traits, *AB* Antisocial behavior

To examine Aim 2, we employed cross-lagged models examining reciprocal associations between CU traits and teacher’s use of reward and harsh discipline. We also included pathways from antisocial behavior at both time points to test the specificity of the relationships between teacher reward and discipline strategies with CU traits. The model also included pathways from the following covariates to variables at both time points: child age, gender, family type, and free school milk. Given that children in the current sample were clustered within one of 11 classrooms, we also accounted for nesting by including dummy codes for each classroom as covariates in both the regression and cross-lagged models. The models were fitted using R software (R Core Team 2013). Since the attrition rate was very low (i.e., 1.8%) in the present sample, selective attrition analyses were not conducted (Fewtrell et al. 2008).

## Results

### Descriptive Analyses

Table 1 presents descriptive statistics for all study variables. The mean scores of CU traits and antisocial behavior, which consist of two fewer items for the antisocial behavior scale to the original measure, indicated that around 15–30% of the current sample evidenced clinically-elevated behavior problems in comparison to prior research in a clinical sample that employed the original UNSW scale (CU traits, *M* = 8.10 and *SD* = 3.33; antisocial behavior, *M* = 10.46 and *SD* = 4.67) (Hawes et al. 2019). Bivariate correlations between main study variables are presented in Table 2. First, child CU traits, antisocial behavior, and teacher rewards and discipline showed moderate stability over time (range, *r =* 0.41–0.59, *p* < 0.01), whereas child academic motivation and school engagement showed relatively high rates of stability over time (*r =* 0.66 and 0.67 respectively, *p*s < 0.01). At both time points, antisocial behavior and CU traits were moderately correlated (Time1, *r* = 0.20, *p* < 0.01; Time 2, *r* = 0.30, *p* < 0.01) and these relationships were also significant longitudinally. There were significant modest correlations between CU traits and teacher use of reward strategies cross-sectionally and longitudinally (range, *r =* −0.31−−0.19, *p*s < 0.01), but harsh discipline at time 1 was only significantly related to CU traits at time 2. On the other hand, antisocial behavior was significantly related to greater use of harsh discipline by teachers at both time points (range, *r =* 0.27–0.37, *p*s < 0.01), but reward strategies at time 1 and time 2 were only significantly related to antisocial behavior at time 2. CU traits were also significantly related to lower academic motivation and school engagement both cross-sectionally and longitudinally (range, *r* = −0.55−0.27, *p*s < 0.01). Similarly, antisocial behavior was significantly related to lower academic motivation and school engagement both cross-sectionally and longitudinally (range, *r* = −0.42−0.22, *p*s < 0.01). Teacher reward strategies were significantly associated with higher motivation and school engagement (range, *r =* 0.16–0.33, *p*s < 0.01), while harsh disciplines were associated with reduced child academic motivation and school engagement (range, *r* = −0.29−0.19, *p* < 0.01) across both time points.

**Table 2:** Cross-sectional and Longitudinal Correlations between Main Study Variables

Variable	1	2	3	4	5	6	7	8	9	10	11	12	13	14	15
1. Gender															
2. Single Parent	−0.01														
3. Free School Milk	0.13*	0.41**													
4. Age	−0.01	−0.10	−0.03												
5. CU Traits Time 1	−0.17*	−0.04	0.06	0.03											
6. CU Traits Time 2	−0.33**	0.04	0.05	0.09	0.46**										
7. AB Time 1	−0.22**	0.13	0.10	0.10	0.20**	0.20**									
8. AB Time 2	−0.21**	0.15*	−0.01	0.05	0.18**	0.30**	0.59**								
9. Reward Strategies Time 1	0.10	−0.04	−0.03	−0.11	−0.20**	−0.20**	−0.07	−0.18**							
10. Reward Strategies Time 2	0.05	−0.07	−0.01	−0.09	−0.19**	−0.31**	−0.03	−0.16*	0.41**						
11. Harsh Discipline Time 1	−0.11	0.03	−0.00	0.21**	0.09	0.16*	0.37**	0.27**	−0.25**	−0.08					
12. Harsh Discipline Time 2	−0.08	−0.11	−0.14*	0.31**	0.08	0.13	0.30**	0.28**	−0.12	−0.11	0.53**				
13. Academic Motivation Time 1	0.15*	−0.07	0.02	−0.05	−0.44**	−0.34**	−0.29**	−0.28**	0.20**	0.20**	−0.19**	−0.21**			
14. Academic Motivation Time 2	0.15*	−0.05	0.02	−0.24**	−0.27**	−0.44**	−0.22**	−0.26**	0.16**	0.21**	−0.27**	−0.27**	0.66**		
15. School Engagement Time 1	0.24**	−0.10	−0.01	−0.13	−0.58**	−0.48**	−0.38**	−0.36**	0.33**	0.31**	−0.29**	−0.27**	0.74**	0.55**	
16. School Engagement Time 2	0.22**	−0.10	−0.01	−0.22**	−0.36**	−0.55**	−0.34**	−0.42**	0.27**	0.33**	−0.27**	−0.29**	0.59**	0.73**	0.67 **

*CU* traits Callous-unemotional traits, *AB* Antisocial behavior. *p < 0.05. **p < 0.01

#### Aim 1: Explore whether CU Traits Moderate the Relationship of Teacher Reward and Discipline Strategies on Academic Motivation and School Engagement

**Fig. 1:**
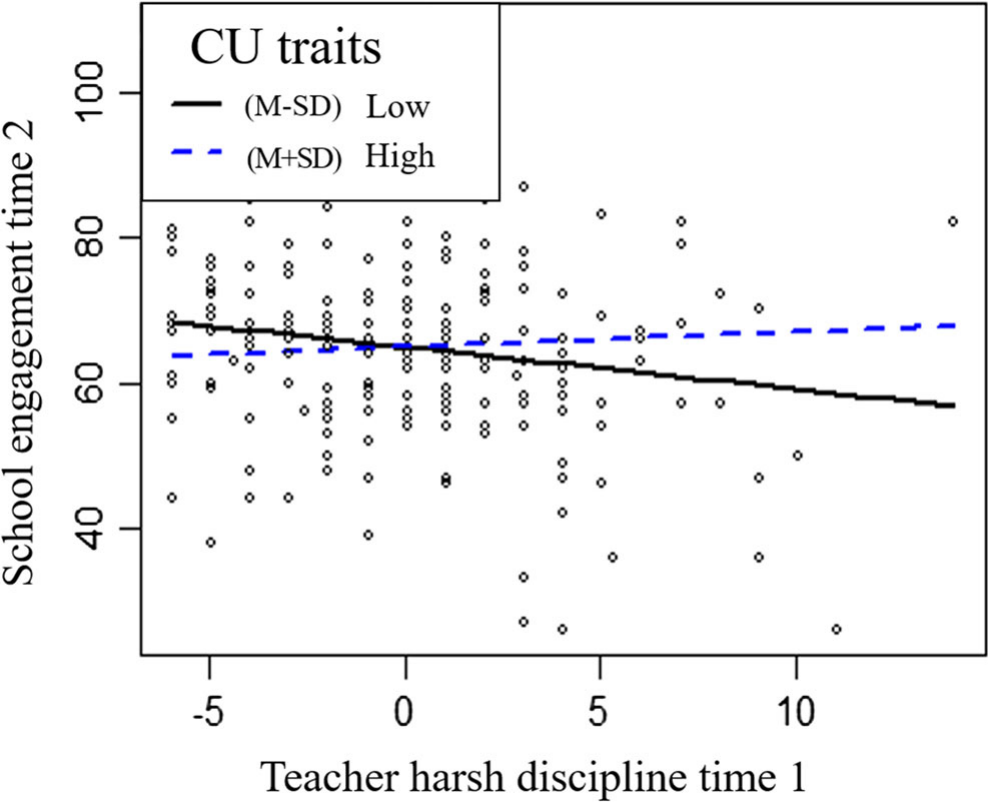
CU = callous-unemotional traits. Longitudinal associations between teacher harsh discipline and school engagement at high (1 SD above the mean) and low (1 SD below the mean) levels of CU traits. CU traits and teacher harsh discipline were centred for interpretation

We found that CU traits moderated the association between teacher discipline, but not reward strategies, and child school engagement (Table 3). Specifically, greater use of harsh discipline by teachers at time 1 predicted lower levels of school engagement at time 2 among children with low (*β* = −0.61, *p* < 0.05), but not high levels of CU traits (*β* = 0.13 *ns*) (Fig. 1). Although antisocial behavior and CU traits were significantly associated with poor motivation and school engagement in bivariate correlations, these were no longer significant after controlling for each other and covariates in the regression model. We did not find any significant main effect of either teacher reward strategies or discipline at time 1 on academic motivation at time 2, and there was no significant interaction effect between teacher reward strategies or discipline and CU traits in predicting academic motivation (Table 3).

**Table 3:** Longitudinal regression analysis testing moderation by CU traits on the association between teacher rewards/harsh discipline strategies and child school related outcomes

Variable	Academic Motivation Time 2				School Engagement Time 2			
	B	SE	β	*p*	B	SE	β	*p*
Age	−1.99	0.95	−0.16	0.037	−3.00	1.53	−0.15	0.053
Gender	0.74	0.89	0.05	0.408	1.09	1.43	0.04	0.447
Family type	−0.96	1.74	−0.03	0.583	−3.25	2.80	−0.07	0.248
Free school milk	0.79	1.71	0.03	0.646	5.45	2.75	0.12	0.048
Antisocial behavior	0.19	0.21	0.06	0.356	−0.32	0.33	−0.06	0.331
Academic motivation time 1	0.70	0.09	0.59	<0.001	0.49	0.15	0.26	0.001
School engagement time 1	0.03	0.06	0.05	0.615	0.38	0.10	0.36	<0.001
CU traits	−0.03	0.19	−0.01	0.868	−0.03	0.30	−0.01	0.911
Reward strategies	−0.11	0.12	−0.05	0.404	0.12	0.20	0.04	0.558
Harsh strategies	−0.20	0.14	−0.10	0.141	−0.21	0.22	−0.06	0.338
CU × Reward strategies	−0.01	0.04	0.00	0.982	0.03	0.06	0.03	0.626
CU × Harsh discipline	0.06	0.04	0.09	0.097	0.13	0.06	0.12	0.035

CU = callous-unemotional traits. CU traits and teacher reward/harsh discipline were centred for interpretation. We created dummy variables for each individual teacher and entered all as covariates in regression model, but not shown in the table

#### Aim 2: Explore whether CU Traits Uniquely Shape Teacher’s Use of Reward or Discipline Strategies over Time

**Fig. 2:**
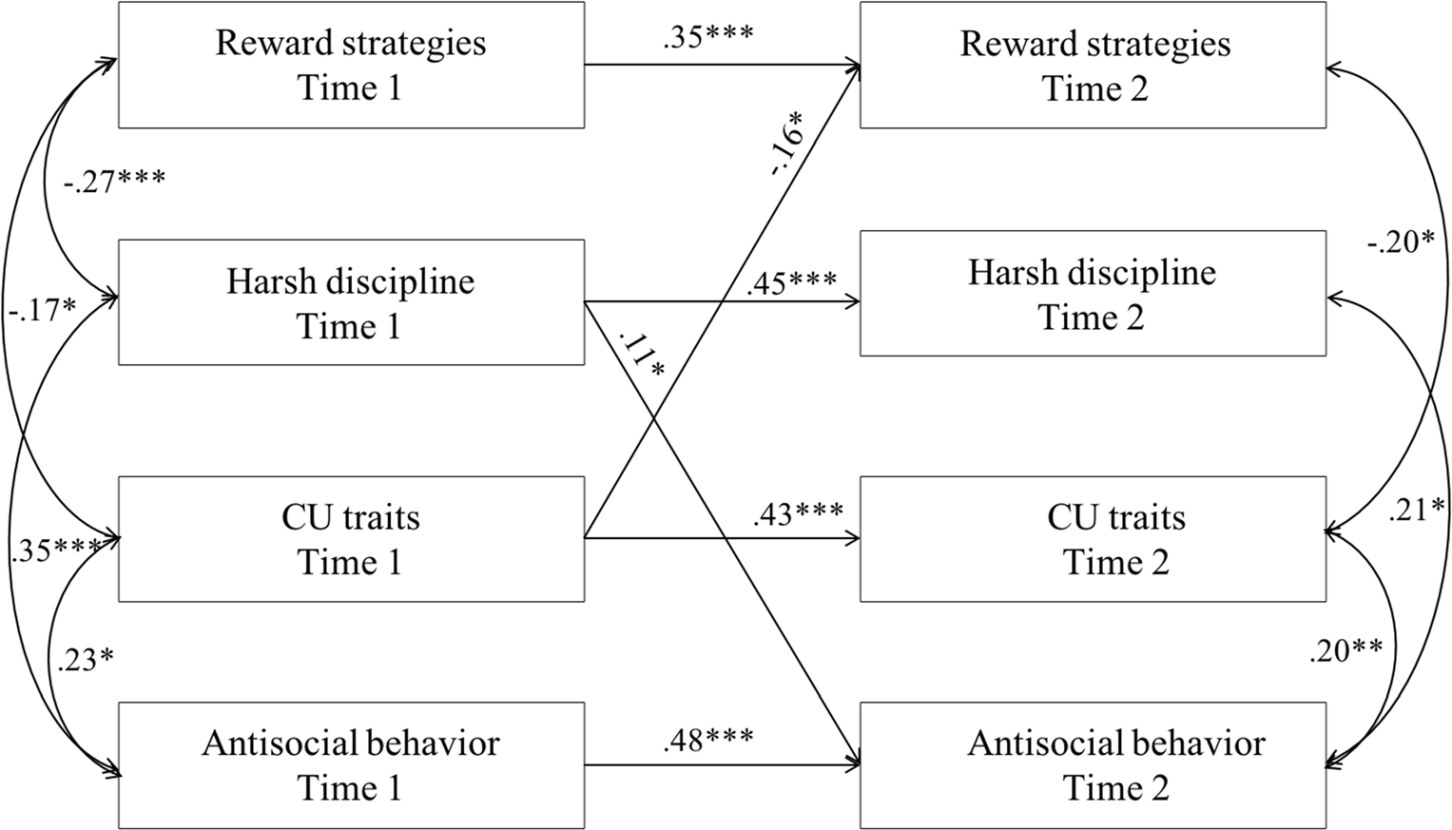
CU traits = callous-unemotional traits. Cross-lagged model between teacher reward and harsh discipline, CU traits, and antisocial behavior at time 1 and 2. Classroom, child age, gender, family type, and free school milk were entered as control variables, but are not shown in the figure. Only significant pathways are shown, the rest of the coefficients are presented in Table S2. * *p* < 0.05. ** *p* < 0.01. *** *p* < 0.001

We found that CU traits uniquely predicted less teacher use of reward strategies over time. Table S2 presents standardized estimates of all pathways between teacher reward and discipline strategies, CU traits, and antisocial behavior. All variables were moderately stable over time (range, *β* = 0.35–0.48, *p*s < 0.001), and there were significant associations between CU traits and antisocial behavior, cross-sectionally at both time points (range, *β* = 0.20–0.23*, p*s < 0.05). In addition, greater use of reward strategies was related to lower CU traits, but not antisocial behavior, cross-sectionally at both time points (range, *β* = −0.17−0.20*, p*s < 0.05). In contrast, greater use of harsh discipline was related to more severe antisocial behavior, but not CU traits, cross-sectionally at both time points (range, *β* = 0.21–0.35*, p*s < 0.05). In terms of cross-lagged effects, higher CU traits predicted less use of rewards by teachers (*β* = −0.16, *p* < 0.05), but not vice versa. In contrast, higher levels of antisocial behavior at time 2 was predicted by greater use of harsh discipline by teachers at time 1 (*β* = 0.11, *p* < 0.05), but not vice versa. Despite significant bivariate longitudinal correlations between harsh teacher discipline at time 1 and CU traits at time 2 (*r =* 0*.*16*, p* < 0.05), this relationship was no longer significant in cross-lagged model that accounted for antisocial behavior, such that CU traits were not significantly related to harsh teacher strategies either cross-sectionally or longitudinally (Fig. 2).

## Discussion

We investigated the moderating effect of CU traits on the link between teacher discipline and rewards on both intrinsic academic motivation and school engagement of 10–12 year old children, as well as longitudinal, reciprocal relations between CU traits and teacher discipline and reward strategies. For the first study aim, our findings supported the hypothesis that CU traits change the impact of teacher harsh discipline on school-related outcomes, even when accounting for antisocial behavior and salient demographic factors, including gender and socioeconomic factors. Teacher’s use of harsh discipline predicted reduced school engagement for children with low, but not high levels of CU traits. Our finding is in line with past research linking CU traits to insensitivity to harsh parental discipline (e.g., Hipwell et al. 2007; Oxford et al. 2003), and qualitative work highlighting teacher’s perspectives on the reduced effectiveness of discipline for children with CU traits (Allen et al. 2016; Allen et al. 2018). Our results extend prior research on CU traits and insensitivity to parental harsh discipline to teacher-child interaction and are consistent with theories emphasizing the role of punishment insensitivity in the etiology of CU traits (Pardini and Frick 2013). On the other hand, our finding is not consistent with prior longitudinal studies reporting that CU traits do not moderate the relationship between harsh parental discipline and antisocial behavior (Waller et al. 2012). Nevertheless, our findings indicate that the contribution of teacher strategies to child school outcomes may be contingent on levels of CU traits. Our longitudinal design advances what is known since prior studies examining the relationships between CU traits and teacher reward and discipline strategies have been cross-sectional and featured small samples (e.g., Allen et al. 2016; Allen et al. 2018).

Our hypothesis that greater use of teacher rewards would predict improved school outcomes specifically among children with high CU traits was not supported. Although teacher reward strategies were not significantly associated with either of the two school-related outcomes regardless of CU traits, it should be noted that teacher rewards were involved in the interaction effect in our model. This may be a potential reason for our findings differing from past research identifying teacher rewards as a means of promoting motivation and engagement in children (Kleinman & Saigh 2011). Another potential reason may relate to the differing cultural context. It may be that the nature and intensity of rewards provided by teachers differs in South Korea. South Korea is a collectivist culture where a high value is placed on interpersonal harmony, meeting social obligations and responsibilities, and the belief that success arises from effort rather than innate ability (Heine and Buchtel 2009). As such, children may be more highly motivated and engaged compared to their Western peers (Shwalb et al. 2009), meaning that external reward contingencies may be more limited in their ability to produce significant gains. Similarly, teachers in Korea may be sparing with praise since work and school tasks are considered the individual’s social obligation and responsibility, producing less variability in the range of reward experiences (Koo 2007). South Korean children may therefore differ in their expectations about, attitudes towards, and exposure to teacher rewards. Further, while treatment research with children referred to clinical services overwhelmingly supports the effectiveness of rewards in reducing antisocial behavior (Leijten et al. 2019), there is also evidence that external rewards can reduce children’s intrinsic motivation and prosocial behavior in community samples (Warneken and Tomasello 2008).

It is also worth considering the complex picture highlighted by qualitative research on CU traits and teacher rewards in relation to the non-significant moderation by CU traits of the purported link between teacher rewards, academic motivation and school engagement. While overall teachers viewed rewards as less effective for children high in CU traits, some also reported iatrogenic effects (e.g., abuse of a position of responsibility), while others identified some reward strategies as helpful (e.g., praise, a positive teacher-child relationship) (Allen et al. 2016; Allen et al. 2018). Past research indicates that CU traits are more strongly related to tangible/monetary rewards and rewards that enhance their status for social dominance compared to those that involve social approval or affiliation (Allen et al. 2016; Foulkes et al. 2014). Future research that delineates the different types of teacher reward strategies may produce a more nuanced understanding of how these strategies relate to CU traits and school outcomes.

The second aim of our study was to investigate direct relationships between CU traits, teacher reward and discipline strategies and antisocial behavior. Our cross-lagged model results partly supported our prediction that CU traits would influence teacher’s use of rewards and discipline, over and above the influence of antisocial behavior. Specifically, we found that higher levels of CU traits, but not antisocial behavior, were related to less use of teacher rewards. Our result is consistent with those of previous cross-lagged studies showing that parenting practices change in response to children’s CU traits, rather than their antisocial behavior (Salihovic et al. 2012; Waller et al. 2014). It may be challenging for teachers to continue to be positive and rewarding towards children with a callous and uncaring interpersonal style. Our prediction that CU traits would increase teacher harsh discipline over time was not supported, in contrast to prior studies that found reciprocal effects between parental harsh discipline and CU traits (Hawes et al. 2011; Salihovic et al. 2012; Trentacosta et al. 2019). Teachers receive training and support in classroom management and have an ethical, legal and professional responsibility to avoid harsh discipline in reaction to child antisocial behavior, whereas parents are not typically exposed to the same level of training, support or scrutiny. Teacher coercive discipline may therefore differ substantially to the discipline of parents in the home, resulting in different child outcomes. Although antisocial behavior did not predict teacher rewards or harsh discipline longitudinally, there were significant cross-sectional associations between antisocial behavior, but not CU traits, and harsh discipline at both time points. Antisocial behavior may be more strongly related to overt disruptive behavior in the classroom, whereas CU traits may be more strongly characterized by deceitful, covert antisocial acts that are less likely to be detected and responded to by teachers in the form of harsh discipline.

Neither teacher rewards nor teacher harsh discipline affected CU traits over time in our study, whereas previous studies examining reciprocal relations between CU traits and parenting practices have supported both child-driven and parent-driven effects (e.g., Trentacosta et al. 2019; Waller et al. 2014). This may be due to the relatively short timeframe between the assessments in the present study or to the differing nature of the relationship between children and teachers compared to that of parents. Clearly the nature, quality and intensity of the teacher-child relationship differs markedly from the parent-child relationship. Another potential explanation relates to age effects. Studies that have found reciprocal effects between CU traits and parenting practices examined young children aged 2–3 years (Waller et al. 2014) or 1.5–4.5 years (Trentacosta et al. 2019), and child characteristics are likely to be more malleable earlier in development. The one study to report only a child-driven effect, without a concomitant parent-driven effect, in linking CU traits and parenting practices focused on adolescents aged 13–15 years (Salihovic et al. 2012). This age range is more in line with our sample of children aged 10–12 years. However, it should be noted that we found a teacher-driven effect in relation to antisocial behavior in the current study, such that greater teacher use of harsh discipline predicted higher levels of antisocial behavior over time. Our findings suggest that teachers react to CU traits rather than antisocial behavior in reducing their reward strategies (i.e., child-driven effect on CU traits), whereas teacher harsh discipline is related to increases in antisocial behavior, but not CU traits (i.e., teacher-driven effect on antisocial behavior). Since all prior studies have examined parenting practices, further studies on teacher-child interactions are clearly warranted to establish the replicability of our findings.

The current findings should be considered in light of several study limitations. First, there may have been important, unmeasured differences between participating and non-participating students (20%), potentially resulting in a biased sample. Second, although we examined the convergent validity between child and teacher report for a subset of the sample, teachers only provided reports on the four children that they identified to show the highest behavior problems, which may have resulted in a restricted range of scores for CU traits or antisocial behavior. However, we wished to ensure we had enough children with problematic levels of both antisocial behavior and CU traits (Muris et al. 2004), so that this subsample would be more representative of children with clinical levels of these difficulties. Children were also the sole informant for the main study variables, introducing the possibility of shared method variance and biases in reporting due to poor quality teacher-child relationships and/or the presence of antisocial behavior and CU traits. In addition, there are concerns about the ability of children to provide reliable responses. However, there is growing recognition that different informants contribute unique information and, as such, children can be an important source of information, especially since adults may not always aware of children’s deceitful or covert antisocial acts (Frick et al. 2010). Future research should strive to include both child and teacher perspectives in addition to classroom observation to overcome these limitations. Third, although we confirmed the construct validity and reliability of a revised version of the UNSW CU traits and antisocial behavior scales using confirmatory factor analysis, the psychometric properties of these measures in South Korean children warrants further investigation. Fourth, there is preliminary evidence that the manifestation and correlates of CU traits may differ in East Asian children compared to Western samples. Fung et al. (2009) found that a sample of Chinese children had higher levels of parent-rated CU traits relative to a sample of US children. Further, Sng et al. (2018) did not find a significant relationship between CU traits and aggression, a construct commonly used to validate measures of CU traits. Evidently, further research is needed to better understand the nature and implications of cultural variation in CU traits in children.

Fifth, it is important to consider that teacher-child interactions are likely more complex and multifaceted than the two dimensions focused on in the current study. For example, prior studies have shown that teachers report greater conflict and less closeness in their relationships with children high in CU traits (Crum et al. 2016; Horan et al. 2016). Thus, the responses of children to reward and discipline strategies may be influenced by a poor-quality teacher-student relationship, which we did not examine in the current study. Sixth, past research suggests that the reward-seeking behavior of children with CU traits may be influenced by the presence or absence of peers, with this influence likely to be heightened during adolescence (Centifanti and Modecki 2013). Indeed, the influence of peers represents a major point of departure between the impact that rewards and discipline might have in the family context compared to the classroom. That is, children are aware of and reacting to the presence of peers during interactions with their teachers, which could moderate the effects of reward or harsh discipline in ways that were not measures in the current study. We used a questionnaire to assess teacher reward strategies, limiting our ability to differentiate between rewards delivered to individual students versus the class as a whole. As children with CU traits are more strongly motivated by self-interest than social connection (Foulkes et al. 2014), individual rewards may carry greater weight than group rewards. The group context of reward receipt is another potential source of cultural variation that warrants investigation, given the emphasis on maintaining ‘face’ in front of others, stronger grounding of the self in social relationships and greater motivation to esteem the group rather than oneself in collectivistic compared to individualistic cultures (Heine and Buchtel 2009).

Our findings indicate that CU traits influence the association between teacher reward and discipline strategies and child school-related outcomes, as well as having a direct effect on the strategies employed by teachers. Children spend significant amounts of time at school from the preschool years onwards, and experience social and academic situations at school that do not occur at home, thus teacher-child interactions may exert unique as well as additive effects on long-term social development. Given that some of our findings differed from the literature on parenting and CU traits (see Waller et al. 2013), it is vital to examine the influence of social interaction on outcomes for children with CU traits in relation to different social roles and contexts. Our findings suggest that teachers will need additional support in implementing discipline and reward-based strategies with children high in CU traits. Future studies should consider additional dimensions of teacher strategies, such as instructional methods influence school-related outcomes for children with CU traits.

## Electronic supplementary material

ESM 1 (DOCX 21 kb)
