# Supplementary material for: Callous-Unemotional Traits and Antisocial Behavior in South Korean Children: Links with Academic Motivation, School Engagement, and Teachers’ Use of Reward and Discipline
Source: J Abnorm Child Psychol. 2020 Jun 16;48(9):1183–95. doi: 10.1007/s10802-020-00663-2 (PMC7392926; doi:10.1007/s10802-020-00663-2)
Supplement: Supplementary file 1 — (DOCX 21 kb) [file 10802_2020_663_MOESM1_ESM.docx]

**Table S1.**

*Factor Loadings for each Item in the Revised Scale, Model Fit Indices and Internal Reliability*

|  | Time 1 | | Time 2 | |
| --- | --- | --- | --- | --- |
| Item | CU | AB | CU | AB |
| SDQ Item 1. Inconsiderate of other people’s feelings | .70*** |  | .75*** |  |
| SDQ Item 3. Does not share with other children | .52*** |  | .48*** |  |
| SDQ Item 5. Unhelpful if someone is hurt, upset, or ill | .75*** |  | .77** |  |
| SDQ Item 9. Not kind to younger children | .49*** |  | .41*** |  |
| SDQ Item 11. Does not volunteer to help others | .70*** |  | .73*** |  |
| APSD Item 17. Not motivated in schoolwork | .54*** |  | .57*** |  |
| APSD Item 21. Breaks promises | .64*** |  | .61*** |  |
| APSD Item 26. No guilt | .26*** |  | .28** |  |
| APSD Item 30. Unconcerned regarding others feelings | .64*** |  | .68*** |  |
| SDQ Item 7. Often fights with other children |  | .39*** |  | .38*** |
| SDQ Item 10. Often lies or cheats |  | .88*** |  | .63*** |
| SDQ Item 13. Steals from home and elsewhere |  | .85*** |  | .52*** |
| APSD Item 15. Blames others |  | .60*** |  | .72*** |
| APSD Item 16. Breaks rules |  | .71*** |  | .60*** |
| APSD Item 18. Acts without thinking |  | .55*** |  | .66*** |
| APSD Item 20. Lies easily |  | .63*** |  | .63*** |
| APSD Item 23. Gets bored |  | .59*** |  | .63*** |
| APSD Item 29. Puts things off |  | .68*** |  | .56*** |
| χ^2^ | 200.70*** | | 174.63* | |
| *df* | 134 | | 134 | |
| *CFI; TLI; RMSEA;SRMR* | .90; .89; .05; .09 | | .95; .95; .04; .09 | |
| α | .73 | .72 | .74 | .68 |

*Note.* CU traits = Callous-unemotional traits; AB = Antisocial behaviour. SDQ = Strengths and Difficulties Questionnaire (Goodman, 1997); APSD = Antisocial Process Screening Device (Frick & Hare, 2001). **p*< .05. ***p*< .01. ****p*< .001.

**Table S2.**

*Unstandardized and Standardized Estimates of all Paths from the Cross-lagged Model*

| Parameter | B | SE | β | *p* |
| --- | --- | --- | --- | --- |
| **Autoregressive Coefficients** |  |  |  |  |
| Teacher Reward Strategies T1 → Teacher Reward Strategies T2 | .35 | .07 | .35 | <.001 |
| Teacher Harsh Discipline T1 → Teacher Harsh Discipline T2 | .47 | .07 | .45 | <.001 |
| CU Traits T1 → CU Traits T2 | .45 | .06 | .43 | <.001 |
| AB T1 → AB T2 | .46 | .09 | .48 | <.001 |
| **Teacher Strategies Predicting Child CU/AB** |  |  |  |  |
| Reward Strategies T1 → CU Traits T2 | -.03 | .05 | -.04 | .560 |
| Reward Strategies T1 → AB T2 | -.07 | .04 | -.11 | .068 |
| Harsh Discipline T1 → CU Traits T2 | .00 | .07 | .00 | .986 |
| Harsh Discipline T1 → AB T2 | .07 | .03 | .11 | .041 |
| **Child CU/AB Predicting Teacher Strategies** |  |  |  |  |
| CU Traits T1 → Reward Strategies T2 | -.21 | .08 | -.16 | .010 |
| CU Traits T1 → Harsh Discipline T2 | .07 | .08 | .05 | .416 |
| AB T1 → Reward Strategies T2 | .12 | .12 | .08 | .296 |
| AB T1 → Harsh Discipline T2 | .17 | .11 | .10 | .119 |
| **Concurrent Covariances** |  |  |  |  |
| Reward Strategies T1 & Harsh Discipline T1 | -4.05 | 1.09 | -.27 | <.001 |
| Reward Strategies T1 & CU Traits T1 | -1.83 | .84 | -.17 | .028 |
| Reward Strategies T1 & AB T1 | -.90 | .74 | -.10 | .220 |
| Harsh Discipline T1 & CU Traits T1 | .91 | .85 | .08 | .282 |
| Harsh Discipline T1 & AB T1 | 3.40 | .81 | .35 | <.001 |
| CU T1 & AB T1 | 1.63 | .78 | .23 | .037 |
| Reward Strategies T2 & Harsh Discipline T2 | -.34 | .74 | -.04 | .646 |
| Reward Strategies T2 & CU Traits T2 | -1.52 | .62 | -.20 | .012 |
| Reward Strategies T2 & AB T2 | -.39 | .42 | -.07 | .357 |
| Harsh Discipline T2 & CU Traits T2 | .26 | .46 | .04 | .258 |
| Harsh Discipline T2 & AB T2 | 1.08 | .42 | .21 | .010 |
| CU Traits T2 & AB T2 | .82 | .29 | .20 | .005 |

*Note.* CU traits = Callous-unemotional traits; AB = Antisocial behavior. We created dummy variables for each individual teacher and entered all as covariates in the model, but these are not shown in the table. Child age, gender, family type, and free school milk were also entered as control variables, but are not shown in the table. T1 = Time 1 assessment; T2 = Time 2 assessment.
